# Mycobacterial DNA-binding protein 1 is critical for long term survival of *Mycobacterium smegmatis* and simultaneously coordinates cellular functions

**DOI:** 10.1038/s41598-017-06480-w

**Published:** 2017-07-28

**Authors:** Shymaa Enany, Yutaka Yoshida, Yoshitaka Tateishi, Yuriko Ozeki, Akihito Nishiyama, Anna Savitskaya, Takehiro Yamaguchi, Yukiko Ohara, Tadashi Yamamoto, Manabu Ato, Sohkichi Matsumoto

**Affiliations:** 1 Department of Bacteriology, Niigata University School of Medicine, 1-757, Asahimachi-Dori, Chuo-ku, Niigata Niigata, 951-9510 Japan; 2 Department of Microbiology and Immunology, Faculty of Pharmacy, Suez Canal University, 41522 Ismailia, Egypt; 3 Department of Structural Pathology, Kidney Research Center, 1-757, Asahimachi-Dori, Chuo-ku, Niigata Niigata, 951-9510 Japan; 4 Biofluid Biomarker Center, Institute of Social innovation and Co-operation, Niigata University, 8050 Ikarashi 2-no-cho, Nishi-ku, Niigata Niigata, 950-2181 Japan; 5 Department of Immunology, National Institute of Infectious Diseases, 1-23-1 Toyama, Shinjuku-ku Tokyo, 162-8640 Japan

## Abstract

Bacteria can proliferate perpetually without ageing, but they also face conditions where they must persist. Mycobacteria can survive for a long period. This state appears during mycobacterial diseases such as tuberculosis and leprosy, which are chronic and develop after long-term persistent infections. However, the fundamental mechanisms of the long-term living of mycobacteria are unknown. Every *Mycobacterium* species expresses Mycobacterial DNA-binding protein 1 (MDP1), a histone-like nucleoid associated protein. *Mycobacterium smegmatis* is a saprophytic fast grower and used as a model of mycobacterial persistence, since it shares the characteristics of the long-term survival observed in pathogenic mycobacteria. Here we show that MDP1-deficient *M. smegmatis* dies more rapidly than the parental strain after entering stationary phase. Proteomic analyses revealed 21 upregulated proteins with more than 3-fold in MDP1-deficient strain, including DnaA, a replication initiator, NDH, a NADH dehydrogenase that catalyzes downhill electron transfer, Fas1, a critical fatty acid synthase, and antioxidants such as AhpC and KatG. Biochemical analyses showed elevated levels of DNA and ATP syntheses, a decreased NADH/NAD^+^ ratio, and a loss of resistance to oxidative stress in the MDP1-knockout strain. This study suggests the importance of MDP1-dependent simultaneous control of the cellular functions in the long-term survival of mycobacteria.

## Introduction

Bacteria belonging to the genus *Mycobacterium* can live for long periods^1–4^. These characteristics appear during mycobacterial diseases, which develop after long-term persistent infections. Mycobacteria comprise two transmissible human pathogens and environmental bacteria that cause opportunistic infections; *M. tuberculosis* (Mtb), the etiologic agent of tuberculosis (TB), which killed more people than any other single infectious agent in 2015 (1.8 million deaths) and *Mycobacterium leprae* which causes leprosy, a disease that results in severe, disfiguring skin sores and peripheral nerve damage. Mycobacteria other than Mtb and *M. leprae* are called non-tuberculous mycobacteria (NTM). Notably, NTM diseases have been increasing and become serious health threats recently^5–7^.

Mycobacterial species can be divided into rapid and slow growers. *M. smegmatis* is a saprophytic fast grower that share the house keeping genes and characteristics of long-term survival with those of pathogenic mycobacteria^8^. *M. smegmatis* is thus used as a model for the study of mycobacterial biology and the long-term persistence^1, 2, 9–12^. However, the fundamental mechanisms of the long-term survival are mostly unknown.

Mycobacterial DNA-binding protein 1 (MDP1), also designated histone-like protein (HLP), HupB, mycobacterial HU, iron-regulated envelope protein (Irep-28), and laminin-binding protein (LBP), is a conserved protein in all examined mycobacterial species^13–17^. MDP1 is a histone-like DNA-binding protein: its N-terminal half shows a similar structure to bacterial nucleoid-associated protein HU^18^, while the C-terminal half contains a large, intrinsically disordered region, which is rare in bacteria. MDP1 suppresses the biosyntheses of macromolecules such as DNA, RNA, proteins and glycolipids *in vitro* ^17, 19, 20^. MDP1 is considered to be essential in slow growing mycobacteria because it is critical in Mtb^18, 21^ (doubling time 15–24 h) and conserved in *M. leprae* (doubling time 2 weeks), the gene-decayed *Mycobacterium*, of which 1,113 genes are pseudogenes^16, 22^. In contrast, MDP1 is dispensable for the rapid grower, *M. smegmatis* (doubling time 3–5 h)^13^.

The expression of MDP1 is enhanced in the stationary growth phase and, in turn, suppresses expression of the catalase-peroxidase KatG, which confers tolerance to isoniazid, a front line TB drug^23^. Isoniazid resistance is one of important markers of mycobacterial dormancy^4, 24–26^. Except in the stationary phase, deficiency of iron, which is an essential metal for the growth, increases MDP1 expression in Mtb^27, 28^. Moreover, oxygen depletion causes growth arrest and induces expression of MDP1 in *M. smegmatis* cultured by Wayne’s dormant model^13^. Taken together, increased MDP1 expression correlates with hostile conditions for multiplication and retarded growth rates. This encouraged us to study the possible role of MDP1 in the long-term survival of mycobacteria.

## Results

### Impacts of MDP1-defeciency on the growth and survival of *M. smegmatis*

**Figure 1:**
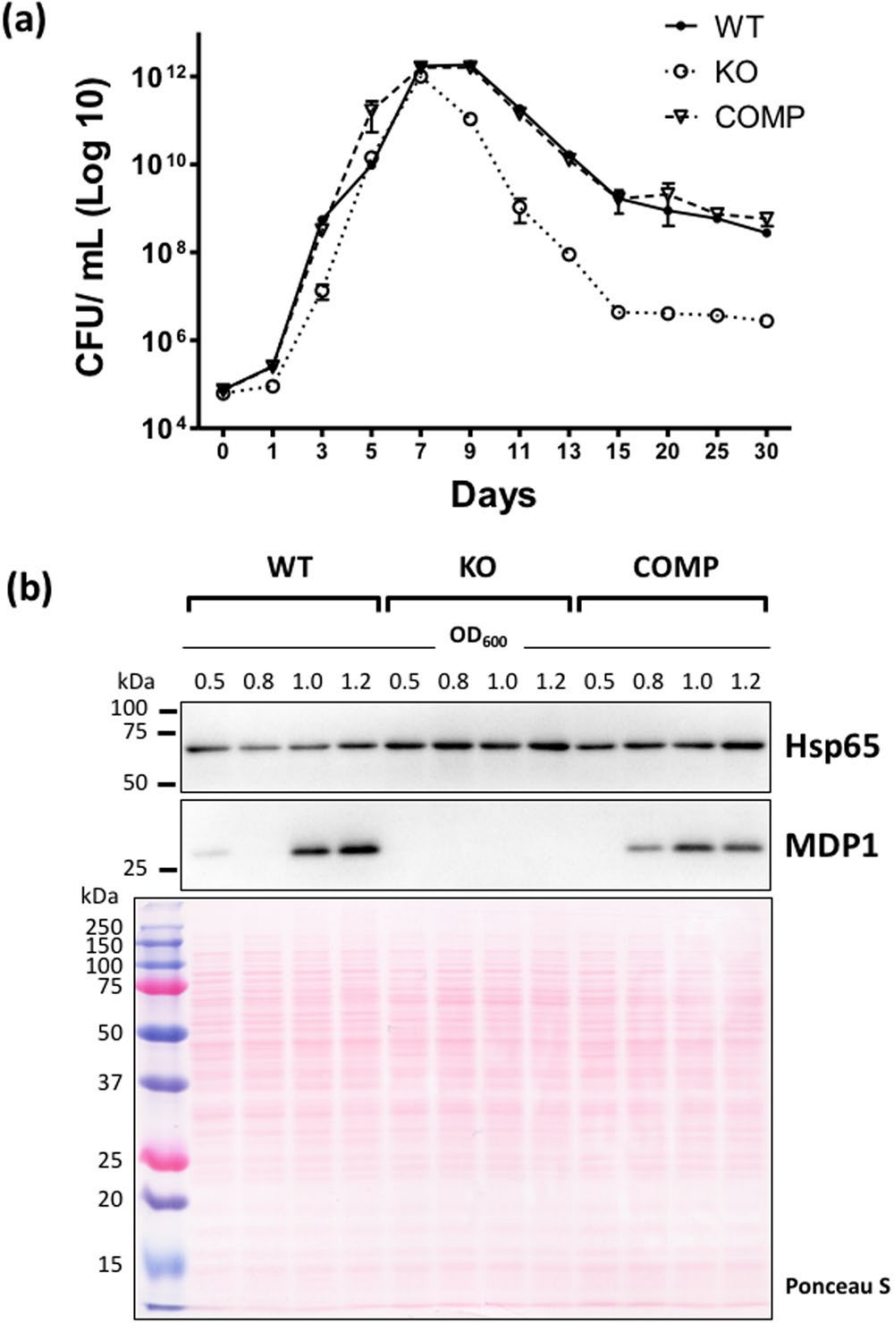
MDP1-KO rapidly dies after entering stationary phase. (**a**) Growth curve of *M. smegmatis* WT, MDP1-KO, and COMP strains over 30 days represented as colony forming units (CFUs)/ml of culture. Bacteria were grown at 37 °C in 7H9-ADC medium with stirring at 60 rpm under normoxia. Data is shown as a representative of duplicate experiments. (**b**) Western blot indicating the expression levels of MDP1 in WT, MDP1-KO (KO), and COMP strains at OD_600_ values of 0.5, 0.8, 1.0, and 1.2. Ponceau S staining of transferred protein on the membrane were shown to confirm the loaded samples have the same amount of proteins and protein bands proved with anti Hsp65 antibody were shown as the internal controls.

To determine the possible role of MDP1 in long-term survival of mycobacteria, we cultured MDP1-defecient *M. smegmatis* (MDP1-KO) in the conventional 7H9-ADC medium under normoxia and compared the growth and survival with those of the parental (wild-type) strain (WT) and MDP1-complemented MDP1-KO strain (COMP). We set the initial viable bacterial number (colony forming units, CFUs) as 7 × 10^4^. As shown in Fig. 1a, MDP1-KO had slightly longer lag phase, but then grew similarly to WT and COMP and entered the stationary phase after 7 days with a CFU count of 1 × 10^12^ ± 0.0003. However, MDP1-KO died more rapidly than the other strains after entering the stationary phase and then reached a plateau after 15 days, maintaining a similar CFU count until Day 30. These data show that MDP1 slightly shortens the lag phase and prevents rapid death of *M. smegmatis* in the stationary and death phases. Consequently, it sustained the number of living bacteria after death phase, during it, the cell count was around 30–1000-fold lower for MDP1-KO than for WT and COMP^29^.

To confirm the upregulation of MDP1 in the stationary phase of *M. smegmatis*, we performed western blot analysis. Anti Hsp65 antibody was used as an internal loading control to confirm that the protein concentrations were equal among samples. Besides we added Ponceau S staining of the proteins prior to antibody reaction in order to check the protein amount of each lane is the same. In the same experimental setting as used to analyze the growth kinetics, we obtained bacteria on Day 6 (OD_600_ values ~0.5 and 0.8, log phase, samples obtained at two different times on Day 6), Day 7 (OD_600_ ~1.0, stationary phase), and Day 9 (OD_600_ ~1.2, stationary to death phase) and monitored MDP1 expression. The amounts of MDP1 at OD_600_ 0.5 and 0.8 were low or below the detection limits but elevated in WT and COMP bacteria with OD_600_ 1.0 and 1.2. The MDP1 expression is higher in WT than COMP. This suggested the expression of MDP1 was partially complemented in COMP strain harboring a single copy of the MDP1 gene inserted in the genome-integration vector pMV306. We confirmed that MDP1 expression was absent in MDP1-KO strain at all the measured data points (Fig. 1b).

### Proteomic analysis shows that MDP1 is a general repressor of gene expression

To determine how MDP1 prevents the rapid bacterial death in the stationary phase, we employed proteomic analysis. Based on the kinetics of growth and the upregulation of MDP1 in the stationary phase (Fig. 1), we extracted proteins from bacteria with OD_600_ = 1.2 and analyzed them. Although we started with an equal culture volume grown to the same OD, the quantification of total protein extracts by BCA assay showed a higher level in the MDP1-KO culture relative to WT and COMP, 740 ± 2 µg, 440 ± 1.7 µg, and 480 ± 1.7 µg, respectively. These data might be consistent with the suppressive activity of MDP1 on the gene expression^17^, although we cannot exclude the possibility that this was caused by the discrepancy between OD values and the real amounts of bacteria. Ten microgram protein extracts from each sample were fractionated by sodium dodecyl sulfate-polyacrylamide gel electrophoresis (SDS-PAGE) (Supplementary Fig. S1a). Densitometric comparison of band intensities between samples revealed a characteristic difference in the fractionation pattern of the MDP1-KO strain, especially between 50 kDa and 75 kDa markers (Supplementary Fig. S1b). We also observed the expected difference in MDP1 itself. Since band differences might be attributable to several proteins with similar molecular weights, we performed more precise identification, using two-dimensional electrophoresis (2-DE) with isoelectric focusing as the first dimension.

Triplicate 2-DE images for a wide isoelectric non-linear range (pI: 3–10) are presented in Supplementary Fig. S2a. Progenesis SameSpot software (Totallab) initially identified 897 dysregulated spots in the MDP1-KO gel images compared to those of WT and COMP. The spot pattern distribution was not well resolved in the acidic part of the gels. To better fractionate the acidic region, we ran additional triplicate gels using a narrower range (pI: 4–7) (Supplementary Fig. S2b). This allowed us to detect 152 more different spots.

**Figure 2:**
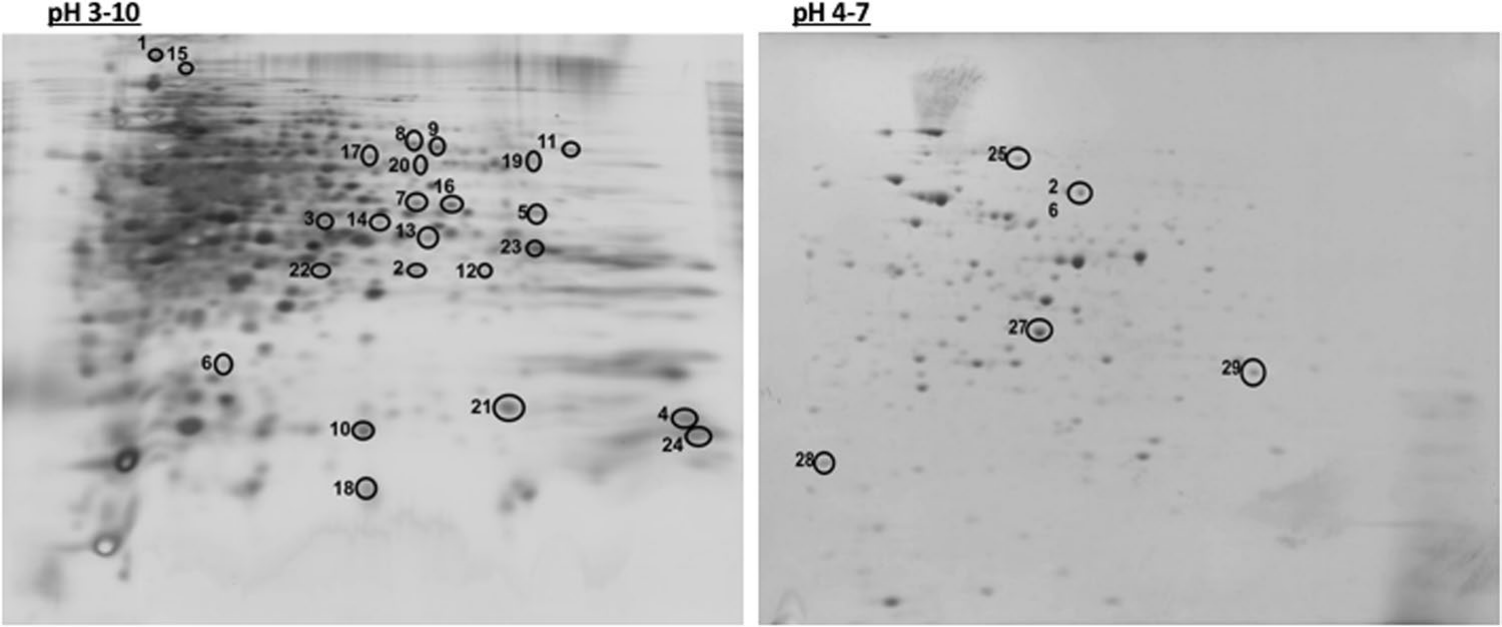
Protein spots over 3-fold-different in MDP1-KO compared with WT. Representative silver-stained two-dimensional gel electrophoresis of the cellular proteins of MDP1-KO (OD_600_, 1.2) and the 29 significantly differentially expressed spots (≥3-fold change) between MDP1-KO and WT gel replicates applied to nLC-MS/MS analysis were marked by circles. The horizontal axis represents isoelectric points (pI) ranging between 3–10 (right) and 4–7 (left), and the vertical axis denotes second dimension electrophoresis by molecular weight.

Initial proteomic analysis of these spots was performed as shown in the chart in Supplementary Fig. S3. Briefly, we applied criteria for filtering the automatically retained results from Progenesis SameSpot software. This step is essential to eliminate biased false positives spots generated from staining artifacts. First, filter 1 (F1) depended on the spot volume intensity to retain spots significantly differ by ≥3 fold. Second, filter 2 (F2) was based on ANOVA to choose spots significantly different between in the WT and MDP1-KO gels (P ≤ 0.05). Third, filter 3 (F3) was a manual filtering step to eliminate suspected background and noise spots. These iterations finally resulted in the identification of 24 and 5 significant spots with over 3-fold difference between MDP1-KO and WT/COMP from the pI 3–10 and 4–7 gel images, respectively (total = 29 spots) (Fig. 2). Interestingly, all the proteins identified bar one were upregulated in the MDP1-KO strain in comparison with both WT and COMP cells. This proteomic comparison confirmed that MDP1 is a general gene expression repressor, as predicted previously^17^.

### Identification of significantly differently expressed proteins due to MDP1-defeciency

We performed mass spectrometric identification of all 29 spots. After removing repetitions, we finally identified 22 proteins (Table 1). Three-dimensional views of intensities of the selected protein spots (spot numbers 1, 9, 11, and 25) are shown in Fig. S4, respectively. These data reconfirmed the previously reported enhanced expression of KatG in the MDP1-KO strain^23^. The effects of MDP1 on the other 21 proteins have not been reported so far.

**Table 1:** Functional identification of the significantly differentially expressed proteins identified by nLC-MS/MS between WT and MDP-1 KO *M. smegmatis* strains.

Spot number	Protein name	Up/Down regulated	Fold change	UniProt Acc No.	Gene symbol	Molecular Function
1	Alkyl hydroperoxide reductase C	Up	4.5	Q57529	*ahpC*	Antioxidant activity
3	Protein RecA	Up	4.4	Q5QJ16	*recA*	Damaged DNA binding
5	Putative oligopeptide permease	Up	4.2	Q938E4		ATPase activity
6	50S ribosomal protein	Down	4.2	O06115	*rplV*	rRNA binding
7	Peptide synthetase	Up	4	Q9RLP6	*mps*	Catalytic activity
9	Chromosomal replication initiator protein	Up	3.9	P0C557	*dnaA*	DNA replication origin binding
10	Putative uncharacterized protein	Up	3.8	B3GNI8		Unknown
11	NADH dehydrogenase	Up	3.8	O52473	*ndh*	Oxido-reductase
12	Beta-lactamase-like protein	Up	3.7	Q9X2P5	*bllp*	Unknown
14	FolD	Up	3.4	Q79N51	*folD*	Methylene-tetrahydro-folate dehydrogenase activity
15	60 kDa chaperonin	Up	3.4	P80673	*groL*	ATP binding
16	DTDP glucose-4,6- dehydrogenase	Up	3.4	Q8GJ79	*rmlB*	Catalytic activity
18	Carbon monoxide dehydrogenase	Up	3.3	Q7X0G4	*coxL*	Oxido-reductase activity
19	Putative oxidoreductase	Up	3.2	Q9AQD0		D-arabinono-1,4-lactone oxidase activity
21	30S ribosomal protein	Up	3.1	P41193	*rpsG*	rRNA and tRNA binding
22	Putative transporter protein	Up	3	Q9RPH4		Transmembrane transport
23	Fatty acid synthetase I	Up	3	Q6XXM0	*fas1*	Enoyl-[acyl-carrier-protein] reductase
24	Integration host factor	Up	4.5	P96802	*mIHF*	Nucleic acid binding
25	Catalase-peroxidase	Up	7	A0A0C4N7T7	*katG*	Catalase activity
26	Acetyl-/propionyl- coenzymeA carboxylase	Up	5.8	A0QTE1	*accA3*	Biotin carboxylase activity
27	Alcohol dehydrogenase	Up	5.5	A0QU52	MSMEG_2079	Oxido-reductase activity
29	Universal stress protein	Up	3.3	A0QZA1	MSMEG_3950	Response to stress

### Enhancement of DNA synthesis on MDP1-defeciency

Several proteins that mediate major cellular functions were included in the protein list which are upregulated on MDP1-defeciency (Table 1). Among them, DnaA is a critical enzyme that initiates DNA replication by binding to a DnaA box near to the replication origin of the bacterial genome and opening it to initiate replication^29–31^. The average normalized abundance of DnaA was significantly higher in MDP1-KO than in WT and COMP (3.9-fold difference) (Fig. S4b). We therefore postulated that MDP1 suppresses DnaA expression and MDP1-deficiency leads to a greater DNA synthesis.

**Figure 3:**
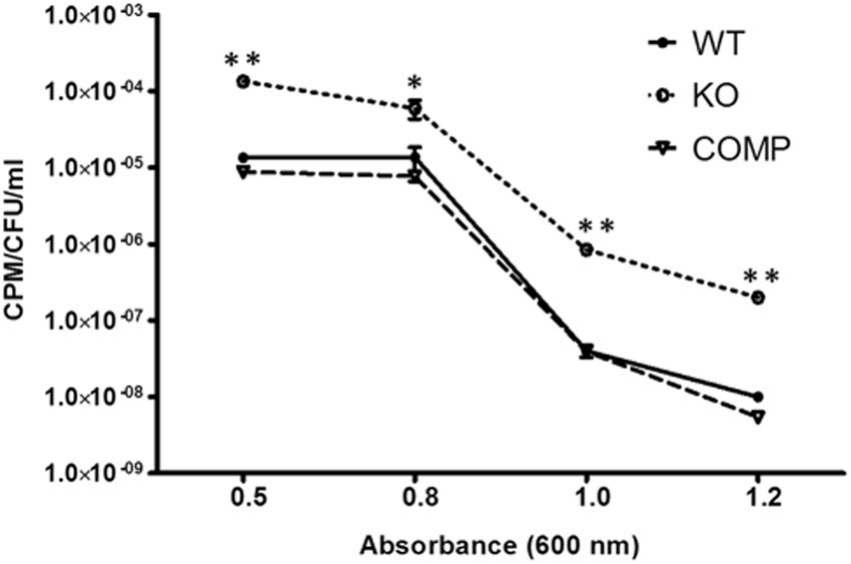
The levels of DNA synthesis in WT, MDP1-KO, and COMP strains. Cultures were grown to OD_600_ values of 0.5, 0.8, 1.0, and 1.2, and synthesis of nucleic acids was chased for 30 min by incorporation of ^3^H-uracil. RNA was hydrolyzed by KOH and the level of DNA synthesis was determined by scintillation counting. The levels of DNA synthesis was expressed as the count of DNA per min per CFU per ml. ANOVA was used to analyze the data and P-values < 0.05 (indicated by *) were considered significant. The experiment was repeated in triplicate.

To address this hypothesis, we chased DNA synthesis in each growth phase, with cells at OD 0.5, 0.8, 1.0 and 1.2, by using ^3^H-labeled uracil and a method established previously^32^. We normalized values by dividing each count per minute (CPM) by its corresponding number of living bacteria (CFU). As shown in Fig. 3, DNA synthesis was higher in bacteria at OD 0.5 and 0.8 (logarithmic growth phase) than in bacteria at OD 1.0 and 1.2 (stationary or death phase). MDP1-KO exhibited elevated DNA-synthesis, demonstrating the suppressive role of MDP1 in replication in the growth and stationary/death phases.

To investigate whether MDP1 controls the transcription of DnaA, we carried out quantitative reverse transcription polymerase chain reaction (qRT-PCR). Expression levels were determined by the comparative threshold cycle method with normalization to those of *sigA* and 16S rRNA. The transcription of *dnaA* was 3.4- ± 1.4 -fold upregulated by MDP1-deficiency, suggesting that MDP1 controls DnaA expression, mainly at the transcriptional level (Supplementary Fig. S5).

### Decrease of NADH/NAD^+^ ratio on MDP1-deficiency

NADH dehydrogenase (NDH) was one of the upregulated proteins in the MDP1-KO strain (Table 1). According to two- and three-dimensional views of the NDH protein spot, the average normalized abundance of this protein was significantly higher in MDP1-KO than in WT and COMP (3.8-fold difference) (Fig. S4c). qRT-PCR analysis showed that the mRNA level was increased 3.6- ± 0.5 -fold in MDP1-KO in comparison with WT (Supplementary Fig. S5); thus, MDP1 controls NDH expression, mainly at the transcriptional level.

**Figure 4:**
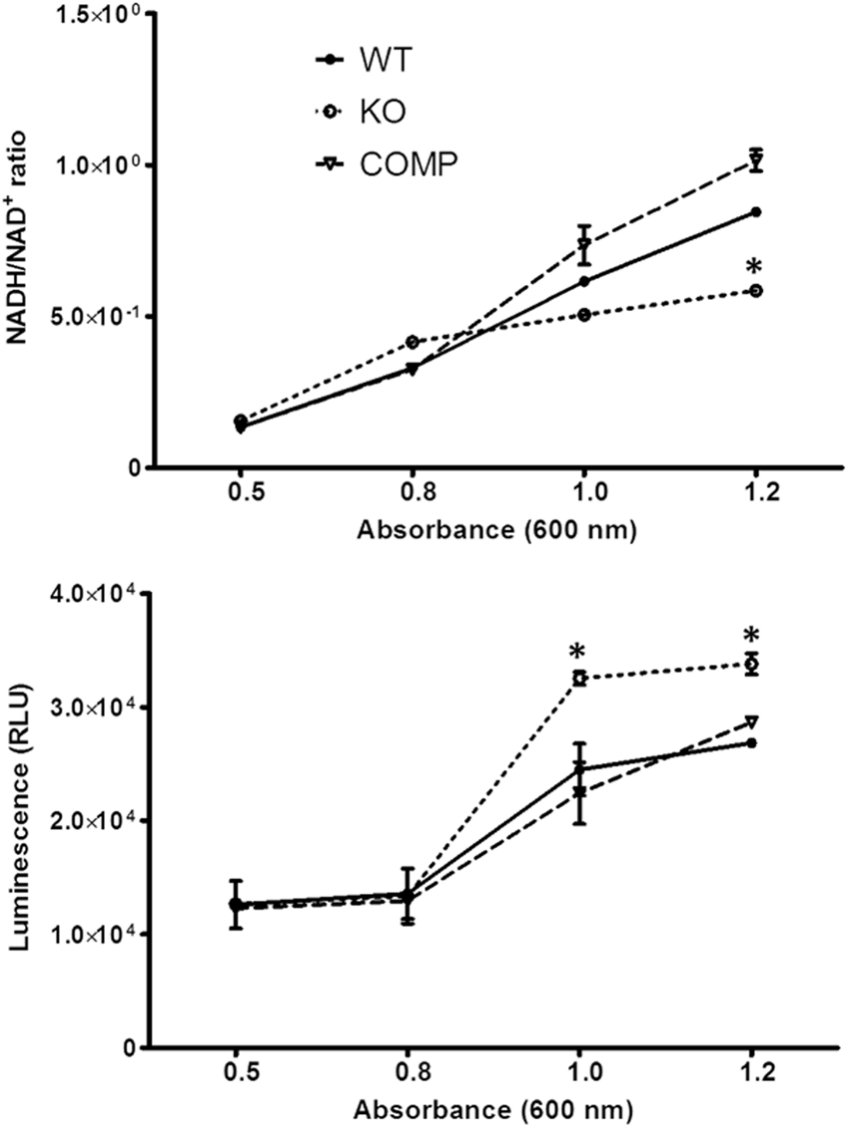
Comparison of intracellular NADH/NAD^+^ ratio and ATP levels in WT, MDP1-KO and COMP. Mean levels of the intracellular NADH/NAD^+^ ratio (**a**) and ATP (**b**) in WT, MDP1-KO and COMP determined by bioluminescence assay. Cultures grew to OD_600_ values of 0.5, 0.8, 1.0, and 1.2. Each experiment was performed with three biological and three technical replicates. ANOVA was used to analyze the data and P*-*values < 0.05 (indicated by *) were considered significant.

Considering the proteomic and transcriptomic data showing upregulation of NDH, which oxidizes NADH into NAD^+^, we hypothesized that the MDP1-KO strain with relatively high NDH activity would contain an altered NADH/NAD^+^ ratio. To test this hypothesis, the intracellular levels of NADH and NAD^+^ were measured in WT, MDP1-KO, and COMP strains at different growth stages (i.e., OD_600_ values) (see Methods) by bioluminescence assay. The NADH/NAD^+^ ratio increased as the culture developed. The NADH/NAD^+^ ratio was found to significantly decrease in the stationary phase of growth in MDP1-KO in comparison with WT and COMP (Fig. 4a, Supplementary Table S1). At OD = 1.2, the NADH/NAD^+^ ratio was 0.59 ± 0.01 in KO and 0.85 ± 0.01 and 1.02 ± 0.04 in WT and COMP, respectively. The ratios at different ODs are summarized in Supplementary Table S1. These data suggested that in the WT, MDP1-dependent inhibition of NDH-expression suppresses NADH dehydrogenase activity in the stationary phase.

### Increase of cellular ATP level on MDP1-defeciency

The NADH/NAD^+^ ratio is a reliable metric that reflects bacterial ATP synthesis^33^. Therefore, we tested the notion that MDP1-KO might alter ATP production. As expected, we found an elevated level of ATP in MDP1-KO cells in the stationary phase compared with WT and COMP (Fig. 4b).

### Reduced resistance to reactive oxygen species (ROS) on MDP1 deficiency

**Figure 5:**
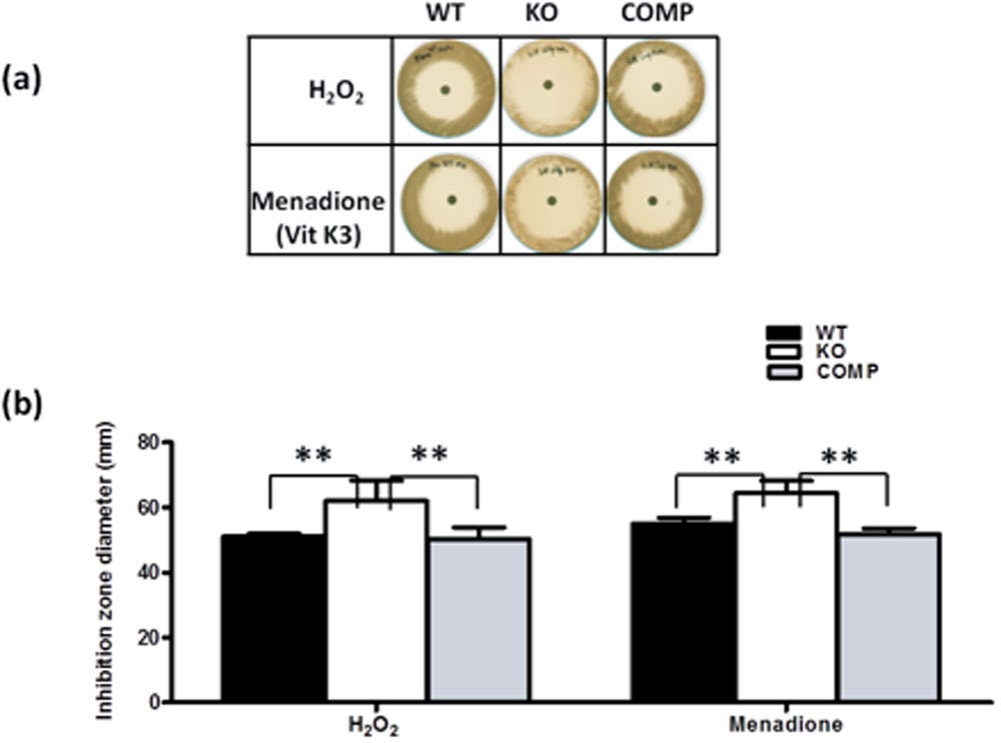
Role of MDP1 in the protection against ROS. Oxidative stress sensitivity assay for WT, MDP1-KO, and COMP *M. smegmatis* strains by analysis on agar soaked with H_2_O_2_ or menadione (Vitamin K_3_). (**a**) Plates showing growth inhibition zones. (**b**) Diameters of inhibition zones measured in mm. Data are shown as means of triplicate experiments. ANOVA was used to analyze the data and P-values < 0.01 (indicated by **) were considered significant.

Expression of two major antioxidant genes of mycobacteria, *ahpC*, alkyl hydroperoxide reductase C, and *katG*, catalase-peroxidase^34–37^, were increased in the MDP1-KO strain in the proteomics analysis (Table 1 and Fig. S4a and d). MDP1 itself is also an antioxidant protein; it has ferroxidase activity and suppresses generation of hydroxyl radicals, the most aggressive ROS, through prevention of the Fenton reaction^38^. Accordingly, there are two general possibilities: either MDP1-deficient *M. smegmatis* becomes more sensitive to ROS through loss of MDP-1, or it becomes more resistant to ROS by upregulation of AhpC and KatG. To determine which, we investigated the effect of growth inhibition by ROS using the disk method^39^. The diameter of growth inhibition produced by exposure to H_2_O_2_ or menadione (vitamin K_3_) was higher in MDP-KO than in WT and COMP, indicating that the MDP1-KO strain is more sensitive to ROS than WT and COMP (Fig. 5). These data demonstrated that MDP1 plays a major role in resistance to ROS.

We confirmed that the expression of *ahpC* and *katG* was mainly regulated at the transcriptional level by MDP1, since qRT-PCR analysis showed that their mRNAs were elevated 6.3 ± 0.4 and 7.6- ± 0.4-fold, respectively in MDP1-KO compared with the WT (Supplementary Fig. S5), although the complemented strain did restore the transcription of the AhpC gene, probably due to the partial complementation of MDP1 in COMP strain.

## Discussion

In the current study, we studied the potential role of MDP1 in the long-term survival of *M. smegmatis*. We showed that expression of MDP1 is increased in the stationary phase^13, 15, 23^ and prevents rapid cell death after bacteria enter this phase, subsequently keeps the surviving numbers of bacteria after death phase, during this time living bacteria acquire nutrients from dead siblings^40^ (Fig. 1). We performed proteomic analysis to elucidate the precise role of MDP1 in prevention of rapid death at stationary to death phases. One of the proteins controlled by MDP1 was DnaA, an initiator of replication. Downregulation of DnaA indicates that *M. smegmatis* actively, rather than passively, terminates replication in stationary phase. This action must be advantageous, since bacteria can save energy by eliminating irrelevant DNA synthesis. Besides interestingly MDP1-dependent suppression of DNA synthesis is not limited to stationary phase (Fig. 3). This implies the involvement of MDP1 in the slow growth itself in log phase, and is consentient with Lewin’s finding that partial reduction of MDP1 expression makes the slow growing *M. bovis* BCG grow faster^20^.

Stationary phase is generally characterized by equilibrium between the numbers of proliferating and dying bacteria, rather than cessation of bacterial growth. However, in this study, the level of DNA synthesis in the stationary phase was as low as <1% of that in the growth phase, even for a similar number of CFUs (1 × 10^11^ ± 0.02 at OD 0.8 and 1.5 × 10^12^ ± 0.04 at OD 1.0 for the WT). This indicates that growth arrest occurred in a large proportion of the bacteria in the stationary phase, rather than being an equilibration of growth and death. The growth status of long term living bacteria without replication is considered to be dormant, implying that dormant bacteria arise in the stationary phase of *M. smegmatis*, as proposed in a previous study^2^.

Proteomic and biochemical analyses further defined the roles of MDP1. We found MDP1 suppressed the expression of Type II NDH, which transfers electrons from NADH to a common quinol pool^33^. Although type II NDH is essential in both *M. smegmatis* and Mtb^41, 42^, it neither translocates protons nor conserves energy, whereas non-essential type I NDH is a proton-translocating enzyme^42^. However NAD^+^ generated by NDH is used in other metabolic reactions to generate ATP. In fact, the ATP level was observed to be higher in the MDP1-KO strain than in the WT and COMP strains (Fig. 4b). There is possibility that the suppression of NDH is involved in the inhibition of ATP production, although it should be clarified by a further study.

Accumulation of toxic metabolites causes bacterial death. ROS are constantly generated as byproducts of aerobic respiration, are generated to a greater extent in stressful conditions including bacterial stationary phases, and are also absorbed from the external environment^43^. Thus, antioxidant defense systems are essential for all aerobic organisms. We found that MDP1 downregulates antioxidant molecules such as AhpC and KatG, while the loss of MDP1 resulted in higher sensitivity to ROS (Fig. 5). This might be explained by the function of MDP1 itself in inhibiting the generation of ROS by preventing the Fenton reaction^38^. Another possible explanation is changed ratio of NADH/NAD^+^, which is important for controlling the redox balance. A lower NADH/NAD^+^ ratio in the stationary phase (Fig. 4) causes a redox imbalance and may cause the higher sensitivity of MDP1-KO to ROS (Fig. 5).

Other molecules controlled by MDP1 have not yet been extensively analyzed, but may also contribute to its function. For example, we found that Fas1, a critical enzyme for cellular fatty acid synthesis, is controlled by MDP1 (Table 1). This implies that MDP1 may suppress membrane and cell wall biosynthesis in the stationary phase by suppression of Fas1 expression.

Taken together, MDP1 has multifunctional properties, such as, suppression of DNA synthesis, respiration, and possibly fatty acid synthesis, and protection against ROS^19^. We consider that MDP1-dependent pleiotropic gene expression control is feasible for long term-survival, because bacteria are able to not only save energy to stay alive but also prevent the generation of toxic metabolites simultaneously. Because MDP1 has iron storage activity^38^ and recently proposed as iron-transporter molecule from ferricarboxymycobactin to mycobactin in the cell envelope of mycobacteria^44^, exhausted iron in the stationary phase might be involved in the loss of viability of MDP1-KO in stationary phase. In this study we used 7H9-ADC medium, which is rich of iron (150 µM). The role of MDP1 related in the iron acquisition should have more impact on the survival of *Mycobacterium* under iron-limited condition, such as, in the macrophages as shown previously^27^. These considerations and reports suggest that MDP1 is a drug target to eradicate persisting mycobacterial pathogens, although further experiments are required to elucidate its role in the pathogenic *Mycobacterium*.

Growing evidence shows that slow growth rate, low metabolism and antioxidant properties are determinants of the longevity in multicellular eukaryotes^45–49^. Interestingly these activities are shared with the functions of MDP1. The present study is likely to provide the novel knowledge to understand the long-term living of the organisms from bacteria to multicellular organisms.

## Methods

### Bacterial strains, culture media, and growth conditions

*M. smegmatis* mc^2^155, *M. smegmatis* mc^2^155Δ*hlp*::pMV306^KM^, and *M. smegmatis* mc^2^155 Δ*hlp*::pMV306^KM^/*hlp* strains were used in this study^23, 50^. Cultures were grown to different ODs according to the experiment, i.e., OD_600_ = 0.5, 0.8, 1.0, and 1.2, in Middlebrook 7H9 broth (Difco) enriched with 0.2% (v/v) glycerol and 0.05% Tween 80 (MP Biomedical). Fifty micrograms per milliliter of hygromycin and 10 µg/ml of kanamycin were added to mutant and complemented mutant strain cultures^50^.

### Measurement of growth kinetics

The growth kinetics of the three tested strains were monitored in terms of CFU per ml for 1 month. On certain days (as shown in Fig. 1), 10-fold dilutions from 10^−1^ to 10^−7^ were made and appropriate dilutions were plated in triplicate on Middlebrook 7H9 agar. The plates were incubated at 37 °C for 5 days before calculating the CFU/ml. The experiment was performed in duplicate.

### Extraction and preparation of bacterial proteins

Mycobacterial cultures were harvested by centrifugation for 10 min at 4,000 × *g* at 4 °C three times. Cellular proteins were prepared from the cell pellets according to Lanigan *et al*. with slight modifications, using bead beating at 4 °C for 30 s intermittently and then adding acetone^51^. Cellular debris was removed by centrifugation and protein lysate was collected and stored at −80 °C. Protein assay of the extracts was carried out using the bicinchoninic acid (BCA) assay kit (Pierce, Thermo Scientific) with bovine serum albumin as the standard^52^.

### SDS-PAGE and western blotting

Seven micrograms of protein extract from each sample were electrophoresed in duplicate on 12.5% SDS-polyacrylamide gels^53^. One gel was stained with Coomassie Brilliant Blue stain (CBB R-250, Wako, Japan) and the second was used for western blotting; the bands were transferred to a polyvinylidene difluoride membrane. For primary antibody incubation, 1:10000 diluted mouse anti-MDP1 antibody (7C monoclonal antibody) was used and anti-heat shock protein Hsp65 (GroEL2) antibody was used as an internal control^23^. Reactions were visualized using ECL Prime western blotting detection reagent (GE Healthcare Lifesciences, Japan). The fractionation patterns were compared using the public domain software ImageJ (a Java image processing program developed by the National Institutes of Health).

### Isoelectric focusing (IEF) and second dimension gel electrophoresis (2-DE)

First dimensional IEF was performed using the Ettan IPGphor 3™ system (Amersham Biosciences, UK) with 7 cm Immobiline™ DryStrips Non Linear pH 3–10 and pH 4–7 (GE Healthcare, UK). Protein extracts were diluted in DeStreak rehydration buffer and focused at 9400 V.h at a maximum voltage of 5000 V. Following IEF, proteins were reduced and alkylated by soaking the strips in equilibration solution (6 M urea, 50 mM Tris-HCl pH 8.8, 30% glycerol, 2% SDS, 0.004% bromophenol blue) containing 10 mM dithiothreitol and 55 mM iodoacetamide^54^. Equilibrated strips were directly used for the second dimension SDS-PAGE as described before^55, 56^. The 2-DE was run in at least in three replicates per sample to ensure maximum accuracy and reproducibility.

### Gel staining, spot imaging and visualization

Gels were stained with silver stain. The 2-DE patterns were recorded as digitalized images and compared using Progenesis SameSpots software (Totallab) for detection of significantly different spots. The software was programmed to select significant spots, ANOVA with P ≤ 0.05 and cutoff spots ≥3-fold different. Spots with significantly altered levels were excised for mass analysis.

### Spot processing and peptide mass mapping by TripleTOF MS/MS

Two-dimensional gels were stained using the Silver Stain Kit for Mass Spectrometry (Piece, Thermo Scientific). The spots of interest were excised and reduced with 10 mM dithiothreitol, carbamide methylated with 55 mM iodoacetamide, and subjected to in-gel trypsin digestion. The peptides from each sample were finally dissolved in 15 μl of 0.3% formic acid, and 5 μl of each sample was injected into a nano-flow-LC (Eksgent nanoLC 415 with ekspert cHiPLC, AB Sciex) coupled with a tandem MS (TripleTOF5600, AB Sciex). Analysis was conducted in duplicate for each sample in trap and elute mode using a ChromeXP C18 Chip column (200 μm × 0.5 mm) as a trap column and a ChromeXP C18 Chip column (75 μm × 150 mm) as an analytical column. Mobile phases A and B were 0.1% formic acid and 0.1% formic acid in acetonitrile, respectively. Peptides were eluted by 20-min gradients from 2% B to 32% B at 300 nl/min. MS spectra (250 msec) followed by 10 MS/MS spectra (100 msec each) were acquired in the data-dependent mode.

### Bioimformatic analysis

Product ion output data were searched against the *Mycobacterium smegmatis* mc^2^155 UniProt KB database (9,855 entries) with concatenated decoy database using a locally stored copy of the Mascot search engine (version 2.4, Matrix Science, London, UK). A protein was accepted if peptides passed the identity and homology thresholds of the Mascot algorithm. The false discovery rate against the decoy database was <1%. Gene Ontology annotation including protein molecular function and biological processes were searched using the SmegmaList database (http://mycobrowser.epfl.ch/smegmalist.html) and UniProt database (http://www.uniprot.org/).

### RNA extraction

Total RNA was extracted from three samples with TRIzol reagent (Invitrogen, Carlsbad, CA) following the manufacturer’s recommendations. Cultures were harvested by centrifugation at 4,000 × *g*. Cells were resuspended in 1 ml TRIzol reagent and disrupted using glass in a Bead Smash 12 (Wakenyaku, Japan). The samples were incubated at room temperature, chloroform was added, and finally RNA pellets were air-dried and redissolved in ultrapure distilled RNase free water (Invitrogen). RNA quantity and quality were assessed.

### Quantitative reverse transcription real-time PCR (qRT-PCR)

To confirm the results of the proteomic findings, qRT-PCR was performed. cDNA was synthesized using the ReverTra Ace qPCR RT Master Mix with gDNA Remover (Toyobo, Japan) per the manufacturer’s instructions. qRT-PCR was performed using THUNDERBIRD SYBR qPCR Mix (Toyobo, Japan) following the manufacturer’s directions. Primers for 6 genes which were designed with Primer3 software as followings: 5′-CGAAGTTGCTGACGTTCACC-3′ and 5′-AAAGTCTGGTCGGCCAATTC-3′ (for *ndh*); 5′-TTGTGTTCGGGTCACATTCG-3′ and 5′-AACCGGTCGTTGTTCATGAC-3′ (for *katG*); 5′AGAAGATGATGCGCCACTTG-3′ and 5′-TTTTGACGATCGGTGACCAG-3′ (for *ahpC*); 5′-TTCAACACGCTGCACAACTC-3′ and 5′-TGATGAGGCCCCATTCGAAC-3′ (for *dnaA*); 5′-CGTTCCTCGACCTCATCCA-3′ and 5′-GCCCTTGGTGTAGTCGAACTTC-3′ (for *sigA*); 5′-TTCAAAGCCGGTCTCAGTTC-3′ and 5′-CGTTGCTGATCTGCGATTAC-3′ (for *16S*). The qRT-PCR reactions were performed using a CFX Connect Real-Time System (Bio-Rad). Relative gene expression was determined from a calculated threshold cycle (CT) and normalized against *sigA* and 16S rRNA as internal standards using their geometric mean following Vandesompele *et al*.^57^. Analysis was performed on at least three biological and technical replicates.

### DNA synthesis measurement by [5, 6-^3^H]-uracil incorporation

DNA synthesis was monitored at different growth points (OD = 0.5, 0.8, 1.0, and 1.2) in each *M. smegmatis* strain in triplicate using 37 kBq of sterile [5, 6-^3^H]-uracil (Moravek Biochemicals) per ml of culture^32^. After ^3^H-uracil incorporation, samples were incubated in 0.3 M KOH at 37 °C for 24 h to degrade RNA from the cells. Cell bound substrates were filtered through 0.45-µm Express Plus membranes (Merck Millipore). Filters were dissolved in 2-ethoxy ethanol then mixed with Ecoscint A scintillation cocktail before counting the radioactivity in a scintillation counter (Aloka liquid scintillation counter, Hitachi). Correction was made for background radioactivity. Viable counts were estimated at each data point by serial dilution. The DNA synthesis was expressed in terms of counts per minute per CFU per ml.

### Determination of NADH to NAD^+^ ratio

Mycobacterial pellets harvested at different OD values were lysed in a basic buffer (0.2 M NaOH and 1% dodecyltrimethyl ammonium bromide). Two aliquots of each sample (100 µl) were taken, one for NADH measurement (base-treated) and the second, mixed with 0.4 M HCl, for measurement of NAD^+^ (acid-treated). Both aliquots were heated at 60 °C for 15 min. Neutralization with 0.4 M HCl/0.5 M Trizma and 0.5 M Trizma was done for base- and acid-treated aliquots, respectively. The assay was carried out using NAD/NADH-Glo™ (Promega) as described by the manufacturer. Luminescence was recorded with a luminometer (Filter Max F5 Multi-Mode Microplate reader) with SoftMax Pro Easy software, and the NADH/NAD^+^ ratio was calculated by comparing the relative light units.

### Measurement of ATP level

Cellular ATP levels were determined in the three tested strains at different OD values by a luciferase bioluminescence assay. Briefly, 100 µl of each sample was mixed with an equal volume of BacTiter-Glo reagent (Promega) in a 96-well plate, incubated for 5 min, and then the luminescence was measured using the previously described luminometer.

### Analysis of oxidative stress sensitivity

Sensitivity to H_2_O_2_ and the superoxide generator menadione were examined by zone inhibition assays, as previously described^39^. Briefly, *M. smegmatis* strains were grown to an OD_600_ of 0.8 in 7H9-ADC medium, and approximately 1 × 10^5^ bacteria were plated onto 7H11- oleic albumin dextrose catalase complex. An 8-mm paper disc saturated with 30 µl of a solution containing 500 mM hydrogen peroxide or 40 mM menadione was placed onto the center of the plate. After incubation for 2 days, the diameter of the zone of growth inhibition generated by each compound was measured.

### Statistical analysis

Data were analyzed using one-way ANOVA and the Kruskal-Wallis non-parametric test followed by Dunn’s multiple comparison test as a post-hoc test. Differences were considered significant when the P-value was ≤0.05.

## Electronic supplementary material

Shymaa et al, Supplement Information

## References

[1] Dick T, Lee BH, Murugasu-Oei B (1998). Oxygen depletion induced dormancy in Mycobacterium smegmatis. FEMS microbiology letters.

[2] Smeulders MJ, Keer J, Speight RA, Williams HD (1999). Adaptation of *Mycobacterium smegmatis* to stationary phase. Journal of bacteriology.

[3] Manabe YC, Bishai WR (2000). Latent *Mycobacterium tuberculosis*-persistence, patience, and winning by waiting. Nature medicine.

[4] Wayne LG, Sohaskey CD (2001). Nonreplicating persistence of mycobacterium tuberculosis. Annual review of microbiology.

[5] Thomson RM (2010). Changing epidemiology of pulmonary nontuberculous mycobacteria infections. Emerging infectious diseases.

[6] Bryant JM (2016). Emergence and spread of a human-transmissible multidrug-resistant nontuberculous *Mycobacterium*. Science (New York, N.Y.).

[7] Namkoong H (2016). Epidemiology of Pulmonary Nontuberculous Mycobacterial Disease, Japan(1). Emerging infectious diseases.

[8] Reyrat JM, Kahn D (2001). *Mycobacterium smegmatis*: an absurd model for tuberculosis?. Trends in microbiology.

[9] Greening C, Berney M, Hards K, Cook GM, Conrad R (2014). A soil actinobacterium scavenges atmospheric H2 using two membrane-associated, oxygen-dependent [NiFe] hydrogenases. Proceedings of the National Academy of Sciences of the United States of America.

[10] Rodionova, I. A. *et al*. Metabolic and bactericidal effects of targeted suppression of NadD and NadE enzymes in mycobacteria. *mBio* **5**, doi:10.1128/mBio.00747-13 (2014). 10.1128/mBio.00747-13 PMC3944813 24549842

[11] Gupta KR, Kasetty S, Chatterji D (2015). Novel functions of (p)ppGpp and Cyclic di-GMP in mycobacterial physiology revealed by phenotype microarray analysis of wild-type and isogenic strains of *Mycobacterium smegmatis*. Applied and environmental microbiology.

[12] Syal K, Bhardwaj N, Chatterji D (2016). Vitamin C targets (p)ppGpp synthesis leading to stalling of long term survival and biofilm formation in *M. smegmatis*. FEMS microbiology letters.

[13] Lee BH, Murugasu-Oei B, Dick T (1998). Upregulation of a histone-like protein in dormant Mycobacterium smegmatis. Molecular & general genetics: MGG.

[14] Prabhakar S (1998). Identification of an immunogenic histone-like protein (HLPMt) of Mycobacterium tuberculosis. Tubercle and lung disease: the official journal of the International Union against Tuberculosis and Lung Disease.

[15] Matsumoto S (1999). Identification of a novel DNA-binding protein from Mycobacterium bovis bacillus Calmette-Guerin. Microbiology and immunology.

[16] Shimoji Y, Ng V, Matsumura K, Fischetti VA, Rambukkana A (1999). A 21-kDa surface protein of Mycobacterium leprae binds peripheral nerve laminin-2 and mediates Schwann cell invasion. Proceedings of the National Academy of Sciences of the United States of America.

[17] Matsumoto S, Furugen M, Yukitake H, Yamada T (2000). The gene encoding mycobacterial DNA-binding protein I (MDPI) transformed rapidly growing bacteria to slowly growing bacteria. FEMS microbiology letters.

[18] Bhowmick T (2014). Targeting Mycobacterium tuberculosis nucleoid-associated protein HU with structure-based inhibitors. Nature communications.

[19] Katsube T (2007). Control of cell wall assembly by a histone-like protein in Mycobacteria. Journal of bacteriology.

[20] Lewin A (2008). The mycobacterial DNA-binding protein 1 (MDP1) from Mycobacterium bovis BCG influences various growth characteristics. BMC microbiology.

[21] Sassetti CM, Boyd DH, Rubin EJ (2003). Genes required for mycobacterial growth defined by high density mutagenesis. Molecular microbiology.

[22] Cole ST (2001). Massive gene decay in the leprosy bacillus. Nature.

[23] Niki M (2012). A novel mechanism of growth phase-dependent tolerance to isoniazid in mycobacteria. The Journal of biological chemistry.

[24] Karakousis PC (2004). Dormancy phenotype displayed by extracellular *Mycobacterium tuberculosis* within artificial granulomas in mice. The Journal of experimental medicine.

[25] Daniel J, Maamar H, Deb C, Sirakova TD, Kolattukudy PE (2011). *Mycobacterium tuberculosis* uses host triacylglycerol to accumulate lipid droplets and acquires a dormancy-like phenotype in lipid-loaded macrophages. PLoS pathogens.

[26] Manina G, Dhar N, McKinney JD (2015). Stress and host immunity amplify *Mycobacterium tuberculosis* phenotypic heterogeneity and induce nongrowing metabolically active forms. Cell host & microbe.

[27] Pandey SD (2014). Iron-regulated protein HupB of *Mycobacterium tuberculosis* positively regulates siderophore biosynthesis and is essential for growth in macrophages. Journal of bacteriology.

[28] Yeruva VC (2006). Identification and characterization of a major cell wall-associated iron-regulated envelope protein (Irep-28) in *Mycobacterium tuberculosis*. Clin Vaccine Immunol.

[29] Mott ML, Berger JM (2007). DNA replication initiation: mechanisms and regulation in bacteria. Nat Rev Microbiol.

[30] Rajagopalan M, Qin MH, Nash DR, Madiraju MV (1995). *Mycobacterium smegmatis* dnaA region and autonomous replication activity. Journal of bacteriology.

[31] Messer W (2002). The bacterial replication initiator DnaA. DnaA and oriC, the bacterial mode to initiate DNA replication. FEMS Microbiol Rev.

[32] Wayne LG (1977). Synchronized replication of Mycobacterium tuberculosis. Infection and immunity.

[33] Tanya Parish, A. B. *Mycobacterium: Genomics and Molecular Biology*. (British Library Cataloguing-in-Publication Data, 2009).

[34] Sherman DR (1996). Compensatory ahpC gene expression in isoniazid-resistant *Mycobacterium tuberculosis*. Science (New York, N.Y.).

[35] Heym B (1997). Effects of overexpression of the alkyl hydroperoxide reductase AhpC on the virulence and isoniazid resistance of *Mycobacterium tuberculosis*. Infect Immun.

[36] Manca C, Paul S, Barry CE, Freedman VH, Kaplan G (1999). *Mycobacterium tuberculosis* catalase and peroxidase activities and resistance to oxidative killing in human monocytes *in vitro*. Infect Immun.

[37] Sherman DR, Mdluli K, Hickey MJ, Barry CE, Stover CK (1999). AhpC, oxidative stress and drug resistance in *Mycobacterium tuberculosis*. Biofactors.

[38] Takatsuka M (2011). A histone-like protein of mycobacteria possesses ferritin superfamily protein-like activity and protects against DNA damage by Fenton reaction. PLoS One.

[39] Pandey R, Rodriguez GM (2012). A ferritin mutant of *Mycobacterium tuberculosis* is highly susceptible to killing by antibiotics and is unable to establish a chronic infection in mice. Infect Immun.

[40] Finkel SE (2006). Long-term survival during stationary phase: evolution and the GASP phenotype. Nat Rev Microbiol.

[41] Miesel L, Weisbrod TR, Marcinkeviciene JA, Bittman R, Jacobs WR (1998). NADH dehydrogenase defects confer isoniazid resistance and conditional lethality in Mycobacterium smegmatis. Journal of bacteriology.

[42] Weinstein EA (2005). Inhibitors of type II NADH:menaquinone oxidoreductase represent a class of antitubercular drugs. Proceedings of the National Academy of Sciences of the United States of America.

[43] Fridovich I (1998). Oxygen toxicity: a radical explanation. The Journal of experimental biology.

[44] Sritharan M (2016). Iron Homeostasis in *Mycobacterium tuberculosis*: Mechanistic Insights into Siderophore-Mediated Iron Uptake. Journal of bacteriology.

[45] Fontana L, Partridge L, Longo VD (2010). Extending healthy life span–from yeast to humans. Science (New York, N.Y.).

[46] Wei M (2009). Tor1/Sch9-regulated carbon source substitution is as effective as calorie restriction in life span extension. PLoS genetics.

[47] Gillooly JF, Allen AP, West GB, Brown JH (2005). The rate of DNA evolution: effects of body size and temperature on the molecular clock. Proceedings of the National Academy of Sciences of the United States of America.

[48] Tolmasoff JM, Ono T, Cutler RG (1980). Superoxide dismutase: correlation with life-span and specific metabolic rate in primate species. Proceedings of the National Academy of Sciences of the United States of America.

[49] Moment GB, Tolmasoff JM, Cutler RG (1980). Superoxide dismutase, thermal respiratory acclimation, and growth in an earthworm, Eisenia foetida. Growth.

[50] Whiteford DC, Klingelhoets JJ, Bambenek MH, Dahl JL (2011). Deletion of the histone-like protein (Hlp) from Mycobacterium smegmatis results in increased sensitivity to UV exposure, freezing and isoniazid. Microbiology.

[51] Lanigan MD, Vaughan JA, Shiell BJ, Beddome GJ, Michalski WP (2004). Mycobacterial proteome extraction: comparison of disruption methods. Proteomics.

[52] Smith PK (1985). Measurement of protein using bicinchoninic acid. Analytical biochemistry.

[53] Enany S, Yoshida Y, Yamamoto T (2014). Exploring extra-cellular proteins in methicillin susceptible and methicillin resistant Staphylococcus aureus by liquid chromatography-tandem mass spectrometry. World journal of microbiology & biotechnology.

[54] Magdeldin S (2014). Basics and recent advances of two dimensional- polyacrylamide gel electrophoresis. Clinical proteomics.

[55] Enany S (2013). Two dimensional electrophoresis of the exo-proteome produced from community acquired methicillin resistant Staphylococcus aureus belonging to clonal complex 80. Microbiological research.

[56] O’Farrell PH (1975). High resolution two-dimensional electrophoresis of proteins. The Journal of biological chemistry.

[57] Vandesompele, J. *et al*. Accurate normalization of real-time quantitative RT-PCR data by geometric averaging of multiple internal control genes. *Genome biology* **3**, RESEARCH0034 (2002). 10.1186/gb-2002-3-7-research0034 PMC126239 12184808

