# Supplementary material for: Mycobacterial DNA-binding protein 1 is critical for long term survival of Mycobacterium smegmatis and simultaneously coordinates cellular functions
Source: Sci Rep. 2017 Jul 28;7:6810. doi: 10.1038/s41598-017-06480-w (PMC5533761; doi:10.1038/s41598-017-06480-w)
Supplement: Supplementary file 1 — Shymaa et al, Supplement Information [file 41598_2017_6480_MOESM1_ESM.doc]

**Supplementary Data**

**Mycobacterial DNA-binding protein 1 is critical for long term survival of *Mycobacterium smegmatis* and simultaneously coordinates cellular functions**

Shymaa Enany, Yutaka Yoshida, Yoshitaka Tateishi, Yuriko Ozeki, Akihito Nishiyama, Anna Savitskaya, Takehiro Yamaguchi, Yukiko Ohara, Tadashi Yamamoto, Manabu Ato, and Sohkichi Matsumoto

| **OD** | **Ratio of NADH/NAD^+a^** | | |
| --- | --- | --- | --- |
|  | WT | KO | COMP |
| **0.5** | 0.14 ± 0.01 | 0.16 ± 0.01 | 0.14 ± 0.01 |
| **0.8** | 0.33 ± 0 | 0.42 ± 0.01 | 0.33 ± 0.01 |
| **1.0** | 0.62 ± 0.01 | 0.51 ± 0.01 | 0.74 ± 0.06 |
| **1.2** | 0.85 ± 0.01 | 0.59 ± 0.01 | 1.02 ± 0.04 |

**Supplementary Table S1. NADH/NAD^+^ ratios in *M. smegmatis* strains in different growth phases**

^a^Values are means ± standard deviations.


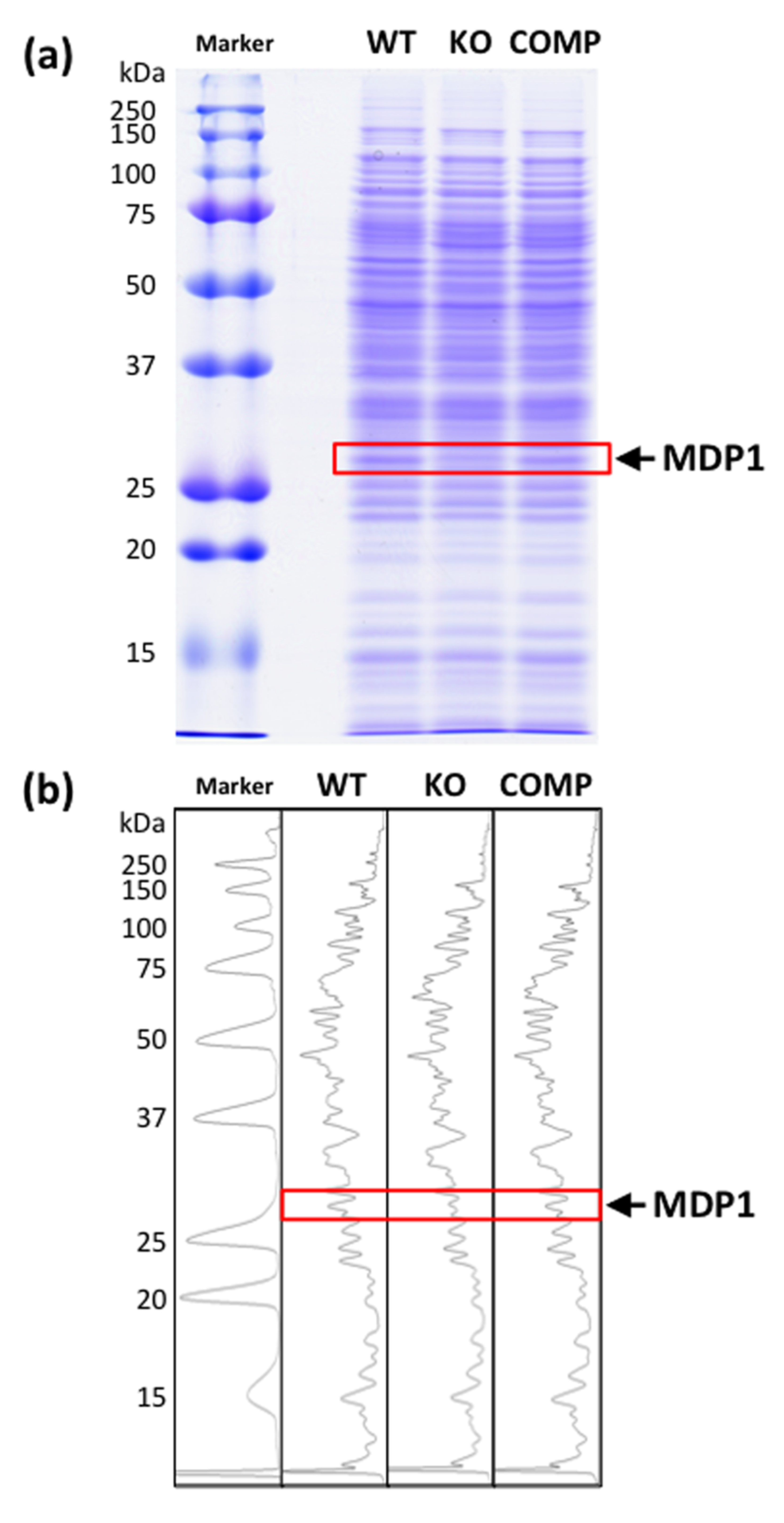


**Supplementary Figure S1. Comparison between protein expression patterns of WT, KO, and COMP strains.**

(a) Coomassie Brilliant Blue R-250 stained gel image of protein extracts of WT (lane 2), MDP1-KO (lane 3), and COMP (lane 4) from cells grown to OD_600_ = 1.2. Samples were run on 12.5% SDS-PAGE; each lane was loaded with 10 µg protein extract. (b). Densitometric comparison of the of protein bands in the extracts of WT, MDP1-KO and COMP strains using NIH ImageJ software. The red boxes in both panels indicates the position of MDP1 at ~27 kDa. Some difference of protein band patterns was found among three strains.


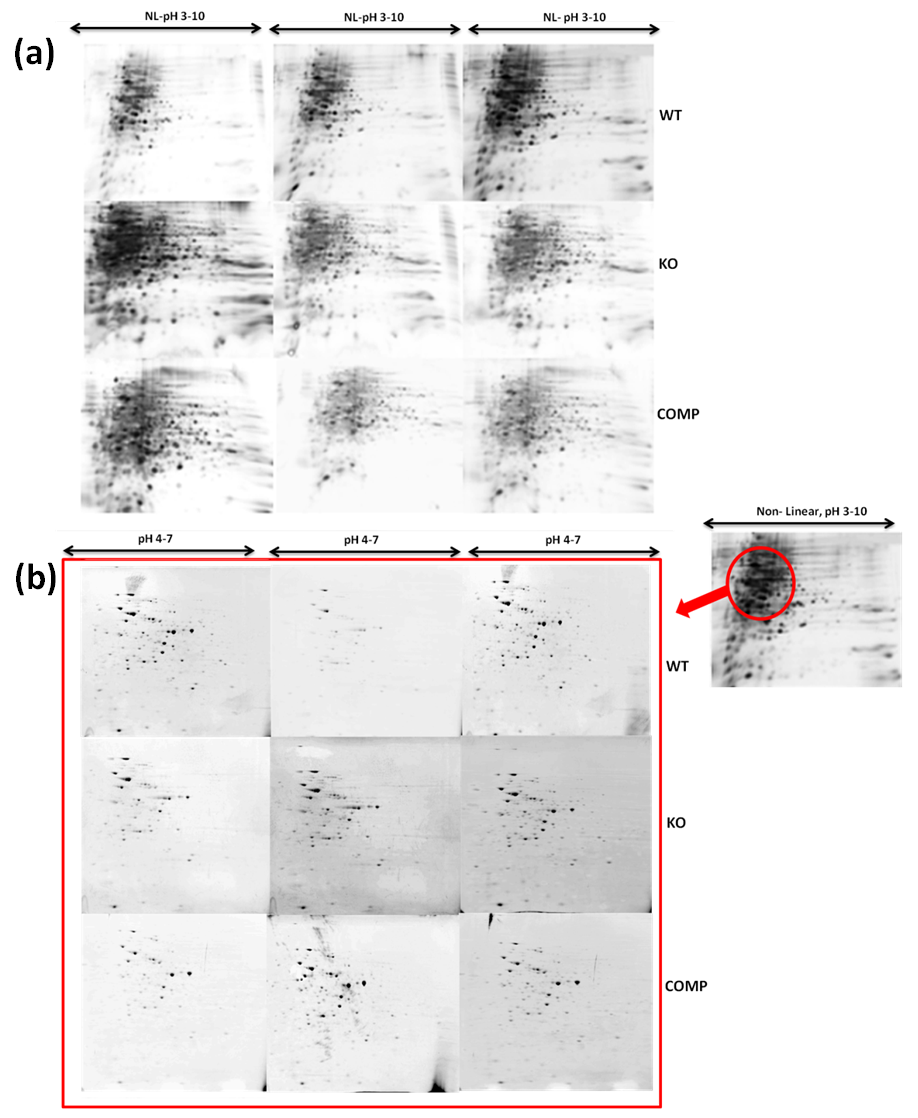


**Supplementary Figure S2. Triplicate sets of two-dimensional gel electrophoresis using Immobilized pH gradient strips**. pH 3–10 NL (non-linear) (a) and pH 4–7 (b) for proteins extracted from WT, KO, and COMP *M. smegmatis*. The horizontal axis represents the isoelectric point (pI) ranging between 3–10 (a) and 4–7 (b), and the vertical axis represents the second-dimension electrophoresis by molecular weight. Gels were silver-stained.


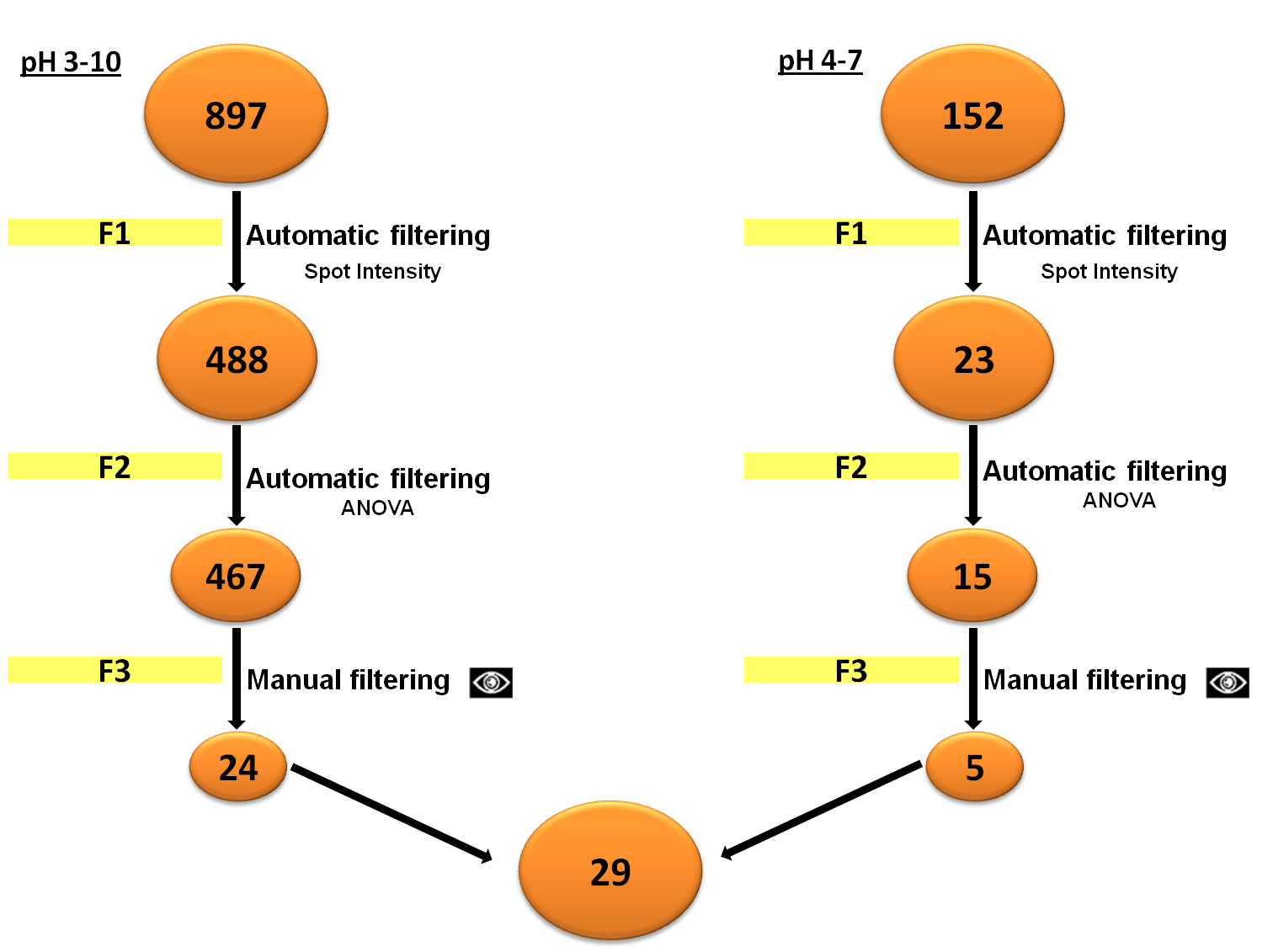


**Supplementary Figure S3. The work-flow of our gel spot refining strategy.** Three filters (two automated and one manual) were used for refining Progenesis SameSpot selected spots. F1 is a spot volume intensity filter to identify spots different by ≥3-fold. F2 is statistical ANOVA filter to identify spots significantly different at P ≤ 0.05. F3 is manual filtering to eliminate suspected background and noise spots. Visualization was achieved by silver staining before spot picking, and was followed by LC-MS/MS analysis of spots.

**
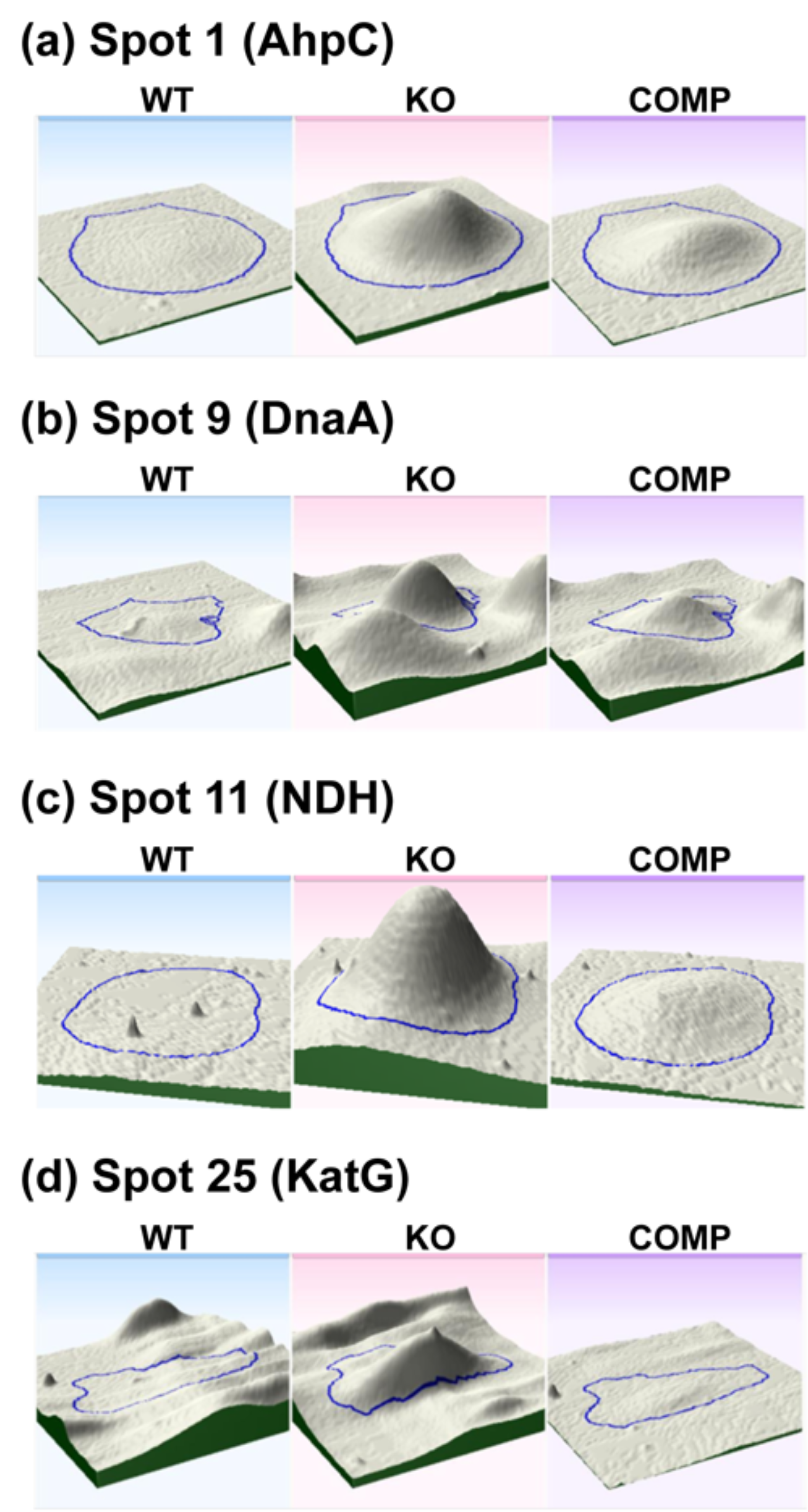
**

**Supplementary Figure S4. Gel spot comparison for differentially expressed Alkyl hydroperoxide reductase C (AhpC), Chromosomal replication initiator protein (DnaA), NADH dehydrogenase (NDH), and Catalase-peroxidase (KatG) proteins between WT (blue), MDP1-KO (pink), and COMP (violet) *M. smegmatis* strains.**

Intensities of gel spots 1 (AhpC, a), 9 (DnaA, b), 11 (NDH, c), and 25 (KatG, d) differentially expressed in 3 strains were compared using Progenesis SameSpots software. 3D views of the intensities of the targeted spot blotted by SameSpots are shown. Spot numbers are corresponding to those listed in Table 1.


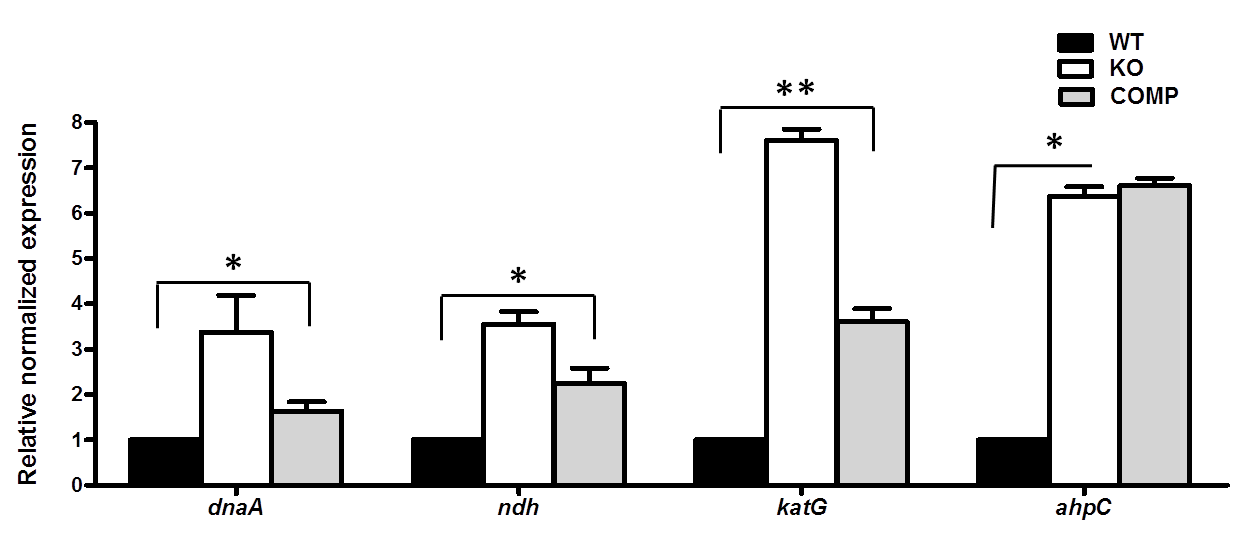


**Supplementary Figure S5.** **Analysis of expression of selected genes in WT, KO, and COMP strains by qRT-PCR.**

Expression of selected genes in WT, MDP1-KO, and COMP were analyzed by qRT-PCR. Cultures were grown to an OD_600_ of 1.0–1.2 and total RNA were extracted from each. mRNA levels were normalized using the geometric mean of the housekeeping genes, *sigA* and 16S rRNA. Data are shown as the means of triplicate experiments with three biological replicates. ANOVA was used to analyze the data and P-values <0.05 (indicated by *) and <0.01 (**) were considered significant.
